# Perceived barriers to compliance with speech-language therapist dysphagia recommendations of South African nurses

**DOI:** 10.4102/sajcd.v67i1.686

**Published:** 2020-09-10

**Authors:** Andrea Robbertse, Alida de Beer

**Affiliations:** 1 Speech, Language, and Hearing Therapy, Medicine and Health Sciences, Stellenbosch University, Cape Town, South Africa

**Keywords:** dysphagia, dysphagia recommendations, compliance, barriers to care, speech therapy, speech-language therapy

## Abstract

**Background:**

Literature has shown that there is limited compliance amongst nurses with the dysphagia recommendations made by speech-language therapists (SLTs). Poor compliance could have a significant impact on the health outcomes of patients with dysphagia.

**Objectives:**

This study aimed to determine the specific barriers to compliance with dysphagia recommendations experienced by South African nurses, with the goal of identifying viable strategies to overcome these barriers.

**Method:**

This cross-sectional study made use of a self-administered questionnaire to obtain quantitative data on nurses’ perceptions of barriers to the implementation of SLT dysphagia recommendations. Eighty-one nurses were recruited from two tertiary hospitals in two South African provinces. Descriptive statistics were used to analyse the reported barriers to compliance.

**Results:**

Three main barriers to compliance were identified, namely a lack of knowledge regarding dysphagia, patient-related barriers and workplace concerns. Knowledge barriers included poor familiarity with the role of the SLT in dysphagia management, lack of knowledge regarding SLT terminology, disagreement with dysphagia recommendations and insufficient dysphagia training. Workplace concerns included staff shortages, heavy workloads and time constraints. Poor patient cooperation was emphasised as a patient-related barrier.

**Conclusion:**

For dysphagia recommendations to be followed by nurses, SLTs need to be aware of the barriers experienced by nurses within the relevant facility. Speech-language therapists need to consider the provision of appropriate in-service dysphagia training and include nurses in the decision-making process when recommendations are made. Speech-language therapists need to consider their role in both clear communication with the nurses and the development of supporting material, such as glossaries and visual aids.

## Background

The incidence and prevalence of non-communicable diseases are rising globally (Institute for Health Metrics and Evaluation, 2018; World Health Organization [WHO], 2014a), along with the sequelae of non-communicable diseases. Dysphagia is a common consequence of non-communicable diseases, including acute neurological fallouts, degenerative conditions, various cancers (Bremare, Rapin, Veber, Beuret-Blanquart, & Verin, 2016; Pace et al., 2009) and trauma-related injuries (Takizawa, Gemmell, Kenworthy, & Speyer, 2016). Poorly managed dysphagia in acute settings could contribute to adverse outcomes such as poor nutrition, dehydration and aspiration pneumonia. Dysphagia subsequently also impacts patients’ quality of life, often resulting in a lack of enjoyment of meals and social isolation if unable to partake in mealtimes as they did pre-morbidly (Dziewas et al., 2017).

Dysphagia management is typically implemented by speech-language therapists (SLTs) and includes the identification, assessment and management of swallowing difficulties, as well as the prevention of related secondary medical complications. In the South African context, however, staff shortages amongst SLTs negatively impact the inpatient care process, with an estimated SLT to South African citizen ratio of 1:25000 (Kathard & Pillay, 2013). Subsequently, SLTs are often dependent on nurses to assist with the implementation of dysphagia management. Nurses are in the unique position to positively influence inpatients’ health outcomes, as they spend the majority of their time interacting with patients (Berry, 2009), allowing for opportunities to monitor swallowing safety and implement SLT dysphagia recommendations.

An interprofessional health model, where healthcare workers of different services provide comprehensive health services for patients, is recommended by the WHO (2010). This approach leads to improved coordination of health services and better patient health outcomes (Dondorf, Fabus, & Ghassemi, 2016). In the South African context, however, interprofessional collaboration is not a consistently viable option because of time constraints, limited access to other professionals and often insufficient coordination between healthcare professionals (Eygelaar & Stellenberg, 2012; Ostrofsky & Seedat, 2016).

According to the nursing scope of practice (South African Nursing Council, 2019), management of dysphagia would fall under ‘implementation of healthcare regimes’ and ‘monitoring of nutritional status’. Nurses’ responsibilities in dysphagia management include administering oral and non-oral feeds, maintaining oral hygiene and ensuring that swallowing techniques and manoeuvres are performed as prescribed by the SLTs.

Nurses are also responsible for providing counselling to patients with dysphagia (Broz, 2012; Chadwick et al., 2013; Jiang, Fu, Wang, & Ma, 2016; Langdon, Lee, & Binns, 2007; Li, Wang, Hang, Lu, & Fang, 2015; Seedat & Penn, 2016).

Colodny (2001) investigated nurses’ compliance with SLTs’ dysphagia recommendations in a high-income country using the mealtime and dysphagia questionnaire (MDQ). Results of this study indicated compliance of less than 50%. Reported barriers to compliance in dysphagia care included a lack of knowledge and training, lack of motivation and disagreement with the SLTs’ recommendations. These findings were supported by Parmelee, Lazlo and Taylor’s (2009) findings, along with staff shortages and lack of physical resources. In the African context, several studies have found similar barriers to compliance (Diendéré et al., 2016; Eygelaar & Stellenberg, 2012; Rhoda & Pickel-Voight, 2015).

General barriers to quality patient care in the South African healthcare environment include staff shortages, time constraints and increased workloads (Eygelaar & Stellenberg, 2012). However, a lack of research relating to barriers associated with dysphagia-specific knowledge and care within the unique South African context was revealed upon review of multiple databases (ScienceDirect, PubMed, EBSCOhost, Sabinet, and Clinical Key).

Although staff shortages are a global concern, these shortages are more severe in South Africa (WHO, 2014b). A 2011 report from the WHO indicated that there are only 40.8 nurses and midwives per 10 000 South Africans. Staffing constraints are particularly prominent in the public healthcare sector, servicing 86% of the South African population (Dondorf et al., 2016; Steyn, Klopper, Coetzee, & Van Dyk, 2015), with only 46% of the nursing workforce being employed in this sector (George, Gow, & Bachoo, 2013). Barriers to care are further exacerbated by the multitude of cultures, languages and religious views that are encountered and need to be considered in healthcare settings. South Africa also experiences a quadruple burden of disease, resulting in medically complex patients needing to be cared for (Kathard & Pillay, 2013). These unique characteristics of the South African healthcare settings can result in increased time spent per patient in an already time-constrained environment.

All of the unique South African characteristics limit the opportunities for interprofessional interaction and further professional development (Eygelaar & Stellenberg, 2012). Given the numerous factors that could hinder quality patient care, queries can be raised about the specific barriers which nurses face when following SLT dysphagia recommendations. Speech-language therapists need to be aware of the barriers to compliance that nurses experience within their respective settings in order to develop meaningful and effective methods of addressing concerns regarding dysphagia management, to ultimately improve service delivery.

Considering the lack of research relating to barriers to compliance in the South African context, the following research question emerged: What are the barriers to compliance with SLT dysphagia recommendations, as perceived by South African nurses in two tertiary hospitals? The study aimed to explore barriers related to: (1) knowledge and training, (2) patients with dysphagia and (3) the working environment.

## Methodology

### Study design and instrumentation

This study followed a quantitative, cross-sectional research design. A revised version of the MDQ, adapted with permission from Colodny (2001), was used to collect data. Adaptations to the MDQ were made to ensure appropriate terminology and phrasing for the South African context, which is considered a low- to middle-income country. For example, ‘resident’ was changed to ‘patient’ to be more representative of the terminology used by South African nurses. Questions were also added to the revised questionnaire to allow for the collection of data relating to dysphagia training received. A pilot study was conducted with 20 nurses with exposure to dysphagia management to ensure content validity and identify ambiguous or superfluous statements. No further changes were made to the revised questionnaire. The revised questionnaire included 28-Likert Scale responses that range from ‘strongly agree’ to ‘strongly disagree’ on statements regarding knowledge and training-related barriers, patient-related barriers and work-related barriers. Facilitators to compliance were also investigated and are being reported in another publication.

## Setting and study population

A total of 81 participants were recruited from two tertiary hospitals (Free State and Western Cape) in South Africa. Convenience sampling was used to ensure access to nurses who were available during the time data were collected. In order to be included, participants needed to be qualified nurses, be formally employed in the public sector and have at least 1 year of experience working with patients with dysphagia.

## Data collection

During the main data collection procedure, potential participants were identified according to the pre-determined inclusion criteria. Informed consent was obtained from all participants after the research and expectations were explained by the researcher. Once the questionnaires were distributed, participants completed them in their own time. The completed questionnaires were then collected within 24 h.

## Data analysis

Descriptive statistics were used to analyse responses obtained, and therefore, no other statistical measurements were utilised. The percentages and frequency for all responses were determined and graphically represented.

## Reliability and validity

A pilot study for the questionnaire was conducted to ensure the reliability and validity of the test instrument. To ensure the validity of this study, sources of bias were reduced by avoiding test effects, as participants had no prior exposure to the testing instrument. To limit reactive effects, data were collected in an unobtrusive manner, by approaching participants in their everyday environment and engaging participants without special equipment, such as microphones. Selection bias was avoided by applying strict inclusion and exclusion criteria, which also increased the generalisability of the study’s findings. Corresponding scores on a split-halves reliability test indicated adequate reliability of the findings.

### Ethical consideration

Permission to conduct the study was obtained from the University of Stellenbosch’s Health Research Ethics Committee (S17/01/014).

## Results

The study samples obtained from both the hospitals were interpreted and described together because of the homogeneity of the participants’ responses. Of the 81 participants, 39 (48%) mentioned their qualifications. Eighteen (46%) were professional nurses, eight (21%) were staff nurses, 12 (31%) were enrolled nursing assistants and one (2%) was a community specialist practitioner. Thirty-three participants (41%) had between 1 and 5 years of work experience, whilst 20 participants (25%) had between 6 and 10 years of experience. Twenty-eight participants (34%) had work experience of longer than 11 years. Table 1 refers to the barriers to compliance with SLT dysphagia recommendations, analysed using descriptive statistics.

**TABLE 1:** Summary of barriers to compliance with speech-language therapists’ dysphagia recommendations (as indicated by the percentage of responses).

Barriers	Specific concerns as indicated by participants	%
Lack of knowledge regarding	The role of the SLT in dysphagia management	46
	Dysphagia terminology	72
	Disagreement with SLT recommendations	45
	Insufficient training regarding dysphagia	72
Patient-related	Uncooperativeness	80
Working environment-related	Staff shortages	91
	Heavy workloads	66
	Time constraints	93

SLT, speech-language therapists.

## Barriers related to training and lack of knowledge

**FIGURE 1:**
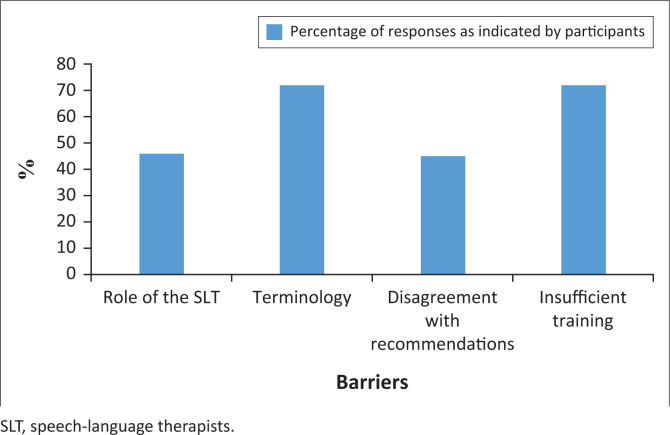
Barriers to compliance related to knowledge and training.

Limited knowledge regarding the role of the SLT in dysphagia management was reported by 46% of participants, whilst 72% of participants indicated poor familiarity with the terminologies used by the SLTs (particularly related to swallowing postures and manoeuvres). Disagreement with the recommendations made by the SLT was reported by 45% of participants. Insufficient training regarding dysphagia management was also reported by 72% of participants. These responses are depicted in Figure 1.

## Barriers related to patients with dysphagia

**FIGURE 2:**
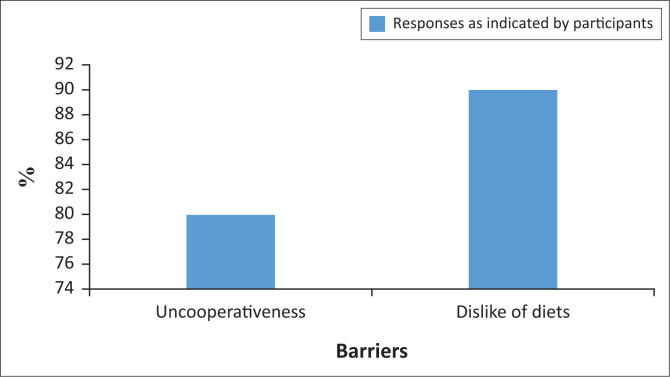
Barriers to compliance related to patients.

Eighty percent of participants indicated that they perceive patients with dysphagia as uncooperative during mealtimes, whilst 90% of participants reported that patients with dysphagia often dislike their meals, leading to poor cooperation and possibly exacerbating other existing barriers to compliance, such as time constraints. These responses are depicted in Figure 2.

## Barriers related to the work environment

**FIGURE 3:**
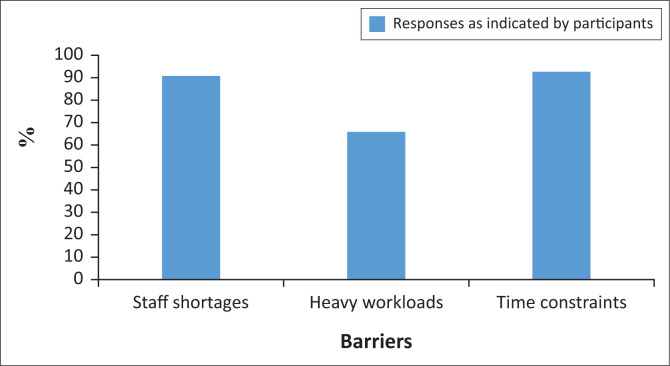
Barriers to compliance related to the working environment.

Significant barriers to compliance in the work environment were noted in this study, specifically staff shortages (91%), heavy workloads (66%) and time constraints (93%). These responses are depicted in Figure 3.

## Discussion

Barriers to compliance were identified in three major themes, namely knowledge and training, patients with dysphagia and the work environment. The knowledge and training barriers identified, included poor familiarity with the role of the SLT in dysphagia management and the terminologies used by the SLTs (particularly regarding swallowing postures and manoeuvres), as well as disagreement with the recommendations made by the SLT. A possible explanation for the barriers identified could be the limited interprofessional contact with SLTs within the nursing environment because of the shortage of SLTs, particularly in public healthcare facilities (Blackwell & Littlejohns, 2010; Pascoe & Norman, 2011). Exposure to other health professions has been reported to increase the transfer of knowledge and skills, leading to overall improved patient care and safety (Berings, Poell, & Gelissen, 2008; Goh, Chan, & Kuziemsky, 2013). Limited contact with SLTs, along with inadequate knowledge of dysphagia management, can contribute to poor familiarity with terminologies used by SLTs. A lack of time allocated to discussing management plans with nurses should also be considered. During this discussion, SLTs could ensure understanding of their proposed dysphagia recommendations and ensure interprofessional agreement. Disagreement with recommendations could stem from nurses’ own opinions, their perceptions of patients’ needs or uncertainty regarding the rationale for specific recommendations (Colodny, 2001).

Barriers to compliance related to patients with dysphagia largely involved lack of patient cooperation. This could result from patients’ medical diagnoses, as well as from personal characteristics such as denial of the presence of dysphagia, dislike of modified diets or limited insights into the rationale for dysphagia recommendations (Horner, Modayil, Chapman, & Dinh, 2016). Variation in patient compliance could also be linked to the diverse demographic of language, cultural aspects and religious beliefs within the context (Riquelme, 2007). Difficulty understanding and expressing oneself in a multi-lingual environment could influence compliance with SLT dysphagia recommendations. Considering barriers to compliance related to patients and the work environment, the observed lack of skills and knowledge amongst nurses potentially influences patients’ health outcomes negatively and could add additional strain for nurses who are already working in stressful environments. Although nurses’ dysphagia knowledge has been investigated in other settings, this is (to the authors’ knowledge) the first study that explored dysphagia knowledge and training amongst South African nurses.

Work environment barriers were characterised by staff shortages, heavy workloads and time constraints. The current work environment, specifically in South Africa’s public healthcare sector, is characterised by a shortage of staff (George et al., 2013; Steyn et al., 2015), because of an insufficient number of undergraduate nursing students, as well as the migration of qualified nurses because of expectations of increased job satisfaction in the private healthcare sector. Staff shortages directly contribute to heavy workloads and subsequent time constraints (George et al., 2013; Steyn et al., 2015). Similar work-related barriers have been noted in both international and African studies. Barriers in the South African work environment are further exacerbated by patient-related barriers. These work-related challenges are often also exacerbated by patient-related barriers mentioned previously, which place further strain on nurses.

Speech-language therapists are not always in a position to directly address issues relating to the work environment and patient-related concerns, especially in the South African context, where a recommendation such as increasing staff numbers is not viable. However, by increasing nurses’ knowledge and training relating to dysphagia management, SLTs can equip them to better manage patients with dysphagia in challenging environments. Training during undergraduate studies and in-service training is recommended to ensure both academic and clinical expertise in dysphagia management.

Interprofessional teamwork between nurses and SLTs is encouraged and needs to include task-sharing and patient discussions on the clinical platform. The WHO (2010) recommends strategies for facilitating collaborative practice between professions and highlights the need for population-specific institutional support, such as shared operating procedures and adequate time and space reserved for interprofessional interaction. A working culture of shared decision-making and routine team meetings is also encouraged.

Interprofessional teamwork will optimise referral systems and reduce time constraints, as referrals to SLTs can be made efficiently. Given that nurses spend approximately 66% of their time in interaction with patients (Berry, 2009; Dondorf et al., 2016), miscommunication and disagreement could be resolved when dysphagia is managed by a collaborating interprofessional team. The nurses can guide the SLTs regarding patients’ personal, cultural or religious preferences. It is recommended that the SLTs consult the nurses when compiling dysphagia recommendations, to reduce disagreement and non-compliance because of a lack of knowledge or feelings of exclusion.

When compiling dysphagia recommendations, patients with dysphagia should be included in the process, as part of patient-centred care. By accommodating patient-specific preferences and needs, realistic and relevant dysphagia recommendations are ensured, thereby potentially improving patient cooperation during mealtimes.

Interprofessional team meetings and in-service training should be scheduled around nurses’ availability to avoid increasing workloads or exacerbating time constraints. Repeating short training sessions in small groups of two to three nurses rather than targeting entire shifts could ensure more efficient and convenient knowledge transfer. Providing nurses written material or visual support as reference could help reduce the need for extended face-to-face contact time during training.

Considering the limited availability of SLTs in South Africa, assistive aids can be used to ensure compliance in the absence of an SLT. Suggestions include visual aids in the form of charts or glossaries. Nurses can then refer to these aids when an SLT is not present to guide the implementation of the dysphagia management recommendations. Reminders on agreed-upon dysphagia recommendations could also be placed next to patients’ beds and in patients’ medical folders. A communication or question book can also be made available for nurses to record questions or comments, which the SLT could then check and respond to regularly.

Within the South African context, it is important for SLTs to recognise their changing role within dysphagia management, beyond direct assessment and treatment. Speech-language therapists need to consider the identification of barriers to compliance and the training of nurses as part of their general patient management. In a context where multiple barriers exist and interact with one another, SLTs should aim to overcome identified barriers to care in their respective healthcare settings.

## Limitations and recommendations

Limitations of this study included the use of convenience sampling, limited demographic information of participants and a lack of context regarding the various nursing qualifications. It is recommended that future studies make use of a random sampling method. Obtaining sufficient demographic information is also recommended to allow for adequate generalisation of findings. Sampling across other sites could also contribute to the generalisation of findings. The use of open-ended questions is also recommended to allow for a more in-depth understanding of participants’ experiences. It is also recommended that future studies investigate the causes of nurses’ disagreement with SLT recommendations.

## Conclusion

This is the first study in South Africa to examine nurses’ perceived barriers to compliance with SLT dysphagia recommendations, with a focus on dysphagia knowledge and training, patients with dysphagia and the work environment. To ensure adequate quality of care and positive health outcomes for patients with dysphagia, recommendations are made to address barriers to compliance. Speech-language therapists need to be aware of nurses’ perceived barriers to compliance within their specific contexts, and attempts should be made to improve inter-professional collaboration, as well as to provide in-service training for nurses regarding dysphagia management.
